# Neuroendoscopy surgery for hypertensive intracerebral hemorrhage with concurrent brain herniation: a retrospective study of comparison with craniotomy

**DOI:** 10.3389/fneur.2023.1238283

**Published:** 2023-09-29

**Authors:** Yuan Zhan, Xiaojun Zou, Jiebin Wu, Liang Fu, Wei Huang, Junming Lin, Fei Luo, Wenhao Wang

**Affiliations:** Department of Neurosurgery, The 909th Hospital, School of Medicine, Xiamen University, Zhangzhou, China

**Keywords:** hypertension, cerebral hemorrhage, brain hernia, craniotomy, neuroendoscopy hypertension, neuroendoscopy

## Abstract

**Background:**

Hypertensive intracerebral hemorrhage combined with cerebral hernia (HIH-CH) is a serious condition. Neuroendoscopy can effectively remove intracranial hematoma, but there is no relevant research support for its utility in patients with HIH-CH. The purpose of this study is to investigate the efficacy and safety of neuroendoscopy in patients with HIH-CH.

**Methods:**

Patients with HIH-CH who received craniotomy or neuroendoscopy treatment were included. The patients were divided into craniotomy (CHE) group and neuroendoscopy (NEHE) group. Clinical data and follow-up outcome of the two groups were collected. The primary outcome was hematoma clearance.

**Results:**

The hematoma clearance rate (%) of patients in NEHE group was 97.65 (92.75, 100.00), and that of patients in CHE group was 95.00 (90.00, 100.00), *p* > 0.05. The operation time and intraoperative bleeding volume of patients in NEHE group were significantly less than those in CHE group (*p* < 0.05). There was no significant difference in the volume of residual hematoma and the incidence of rebleeding between the two groups (*p* > 0.05). The length of stay in ICU in NEHE group was significantly shorter than that in CHE group (*p* < 0.05).

**Conclusion:**

Neuroendoscopy can safely and effectively remove the intracranial hematoma in patients with hypertensive intracerebral hemorrhage and cerebral hernia, significantly shorten the operation time, reduce the amount of intraoperative hemorrhage, shorten the ICU stay.

## Highlights

**- What is already known on this topic** – As drainage and craniotomy are the main treatments for hypertensive intracerebral hemorrhage with - cerebral hernia (HIH-CH), there is a lack of relevant research for the support of utility of neuroendoscopy in these patients.
**- What this study adds** – Neuroendoscopy can safely and effectively remove the intracranial hematoma in patients with hypertensive intracerebral hemorrhage and cerebral hernia.
**- How this study might affect research, practice or policy** – In future clinical practice and study, neuroendoscopy should be a reasonable choice for patients with HIH-CH.

## Introduction

Hypertension is a common disease in the world, which brings huge burden to human beings and causes about 10 million deaths every year (1). Epidemiological data show that there were about 1.39 billion hypertensive patients worldwide in 2010 (2). China is a large country of hypertension. According to previous studies, the prevalence of hypertension among Chinese adults (≥ 18 years old) is 27.9%, and the number of adult hypertensive patients is 245 million (3). Hypertensive intracerebral hemorrhage is one of the serious complications of hypertension, accounting for 28% of all stroke in European and American countries and 48% in China (3, 4). Cerebral hernia is one of the serious manifestations of hypertensive intracerebral hemorrhage, which can lead to secondary brain stem injury with high mortality and poor prognosis (5, 6). At present, drainage and craniotomy are the main treatments for hypertensive intracerebral hemorrhage with cerebral hernia (6). In recent years, neuroendoscopy has been gradually applied to the treatment of hypertensive intracerebral hemorrhage, and achieved good results (7–9). Neuroendoscopy can clearly explore the intracranial structure, and at the same time, it has a certain amplification effect, that is, large lesions in the brain can also be removed through a small incision (10, 11). Endoscopic neurosurgery can reduce the risk of bleeding and infection during operation. Endoscopic neurosurgery is also performed under direct vision, which can effectively protect brain tissue by reducing the stretch and damage to brain tissue. Nishihara et al. showed that compared with hematoma puncture and drainage, neuroendoscopy can remove hematoma faster, relieve the compression of hematoma on surrounding normal brain tissue, reduce the occurrence of brain edema, and shorten the length of stay in ICU (10, 12). Some previous studies have shown that endoscopic neurosurgery is superior to microsurgery in terms of intraoperative bleeding, hospital stay, and lung infection rate, and NHISS score is superior to craniotomy, which can significantly reduce the disability rate and mortality of patients (13, 14). However, there is a lack of relevant research for the support of utility of neuroendoscopy in hypertensive intracerebral hemorrhage combined with cerebral hernia. The purpose of this study is to investigate the efficacy and safety of neuroendoscopy in patients with hypertensive intracerebral hemorrhage combined with cerebral hernia through preliminary retrospective analysis, so as to provide a reference for deciding whether to use neuroendoscopy in clinical practice and for future research.

## Methods

### Study population

Hypertensive cerebral hemorrhage patients admitted to our department from January 2015 to June 2021 were enrolled. Inclusion criteria: (1) Age ≥ 18; (2) All cases were confirmed as supratentorial hemorrhage by CT; (3) Clinical physical examination showed dilated pupil on one side; (4) acute onset hemorrhage and emergency operation was performed; (5) Follow up for more than 3 months. Exclusion criteria: (1) Bilateral mydriasis; (2) Complicated with malignant tumor, rheumatic diseases, arteriovenous malformations, aneurysms, moyamoya disease, severe cardiopulmonary disease, diabetes, renal insufficiency and coagulation dysfunction; (3) Incomplete follow-up data.; (4); (5) In this study we included cerebral hernia confirmed by midline shift on CT and ipsilateral large fixed pupil. This study was approved by the Ethics Committee of the 909th Hospital, School of Medicine, Xiamen University (Approval number: L2022011). At the same time, as a retrospective study, patients were exempted from signing the informed consent form. This study strictly follows the STROBE statement and its checklist. After the patients were included in the final analysis, they were divided into Neuroendoscopic hematoma evacuation group (NEHE group) and Craniotomy hematoma evacuation group (CHE group).

### Treatment

All cases underwent emergency operation under general anesthesia. The bedside head CT was reviewed routinely within 3 h after operation. All patients were treated with early rehabilitation.

#### Neuroendoscopic hematoma evacuation

**Figure 1:**
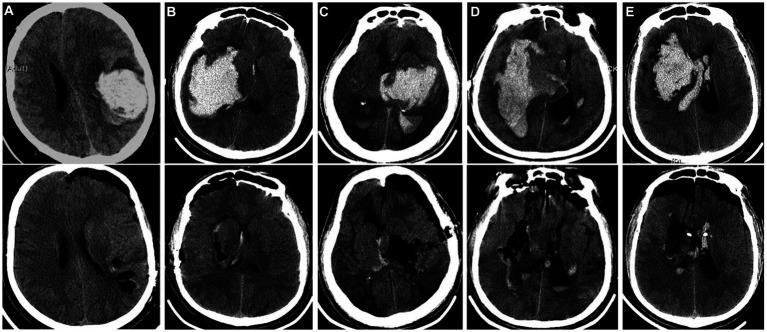
CT comparison of typical cases of hypertensive intracerebral hemorrhage before and after endoscopic clearance. **(A)**: Simple lobar hematoma. The hematoma almost breaks out of the cortex. Bedside CT locates the shallowest part of the hematoma as the puncture point, and a channel was puts to remove the hematoma; **(B)**: The amount of hematoma in the basal ganglia area is large, and the hematoma has reached the temporal lobe cortex. The bedside CT located the shallowest part of the hematoma as the puncture point, and a channel was placed to remove the hematoma; **(C)**: The hematoma in the basal ganglia and thalamus breaks into the ventricles of the brain. The hematoma extends from the cortex to the thalamus. The longest diameter of the hematoma is to clear the hematoma through the temporal lobe fistula. The channel does not need to swing obviously, and the damage is small; **(D)**: The giant fusiform hematoma in the basal ganglia was removed by navigation via the long frontal axis approach; **(E)**: The hematoma in the basal ganglia area broke into the ventricle, and the channel was placed through the puncture point of the ventricle under the guidance of navigation. At the same time, the hematoma in the basal ganglia area and part of the hematoma in the ventricle were cleared.

**Figure 2:**
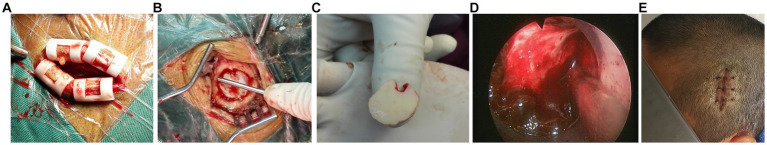
Endoscopic clearance of hypertensive intracerebral hemorrhage assisted by small bone window. **(A)**: The small incision is about 4 cm long; **(B)**: The diameter of small bone window is about 2 cm; **(C)**: Small bone flap removed by milling cutter; **(D)**: Clear passage fistulation and endoscopic evacuation of hematoma; **(E)**: Healing incision.

Before operation, the thickest section of hematoma and the puncture point nearest to the body surface should be located by conventional bedside CT, and the functional area should be avoided from puncture access. A 3.5 ~ 4.5 cm incision was made with the puncture point as center. According to the size of the hematoma, a round bone window with a diameter of 2.0 ~ 2.5 cm was made. After suspension and incision of the endocranium, the dura mater was opened, then the cortex was punctured with a self-made transparent endoscopic channel under the monitoring of the endoscope, and the hematoma was cleared under the direct vision of the endoscope after reaching the hematoma cavity. If there is obvious bleeding, unipolar electrocoagulation and aspirator were used to stop bleeding. Hemostatic gauze, gelatin sponge or fluid gelatin can be used to stop bleeding if there is a little bleeding in the cavity wall of hematoma. If the hematoma breaks into the ventricles of the brain, a drainage tube shall be placed at the hematoma site after the operation (when no bleeding is found on the CT of the head after the operation, the drainage tube shall be removed within 3 days). Then the channel was pulled out and the bone defect was repaired. At the end of surgery, the skull cavity was closed tightly and the scalp tissue was sutured carefully. The specific operation process is shown in Figure 1, and the perioperative CT images of typical cases are shown in Figure 2.

#### Craniotomy hematoma evacuation

Craniotomy was performed with bone flap under microscope. A horseshoe shaped incision was made routinely, and the bone flap was formed with a milling cutter. After opening the dura mater, the cortex was separated from the hematoma with the brain pressing plate, and the hematoma was removed under the microscope. If the hematoma breaks into the ventricles of the brain, a drainage tube shall be retained at the hematoma site after surgery (when no bleeding is found on the CT of the skull after surgery, the drainage tube shall be removed within 3 days), bone flaps shall be removed, a drainage tube shall be retained subcutaneously, and the skull cavity shall be closed routinely and scalp tissue shall be sutured.

### Outcome

The primary outcome of this study was hematoma clearance rate. Calculation method: hematoma clearance rate = (preoperative hematoma volume - postoperative residual hematoma volume) / preoperative hematoma volume × 100%. Hematoma volume was calculated with 3D Slicer software. The secondary outcomes of this study were the operation time, intraoperative bleeding, postoperative bleeding, massive cerebral infarction, Glasgow Outcome Scale (GOS) score at 3-month follow-up, hospital stay and ICU hospital stay, and the incidence of adverse events during the follow-up period.

### Follow-up

All patients received regular long-term follow-up after surgery, and the scheduled follow-up time was 1 month, 3 months. If there is any change in the condition beyond the scheduled follow-up, the patients can visit outpatient clinic at any time. Each follow-up includes but is not limited to: blood cell count, regular urine and stool test, liver function, kidney function, coagulation function, brain CT, GOS score, National Institute of Health stroke scale (NIHSS) score, Activities of daily living (ADL) score and Quality of Life (QOL) score. The NIHSS, ADL and QOL score were obtained in outpatient clinic by physicians (ZY, ZX, WJ, FL, and WH). Adverse events during the follow-up period included all cause death, brain related death, recurrent intracranial bleeding, ischemic stroke, acute myocardial infarction, gastrointestinal bleeding, etc.

### Data collection

All data of this study were extracted based on the electronic medical record system, including baseline data, namely, demographic information, including age, sex, history of cigarette or alcohol use, medical history, preoperative physical examination information, including height, weight, systolic blood pressure, diastolic blood pressure, consciousness. The operation time, intraoperative bleeding volume, residual hematoma volume, recurrent postoperative ebleeding and secondary massive cerebral infarction were all recorded and collected. Severe disability in GOS score was defined as: The patient is conscious, but their function is extremely limited and requires long-term care. Massive cerebral infarction was defined as the diameter of the infarct is>3 cm and involves more than 2 anatomical areas, or the infarct area is>20 cm^2^ and involves more than 2 anatomical areas (15). Ultra-early surgery was defined as hematoma surgery was conducted within 4 h after onset of stroke.

### Statistical analysis

SPSS 24.0 statistical software (IBM, United States) is used for statistical analysis. Continuous variables were represented by median or mean ± standard deviation, and Wilcoxon rank sum test or student t test was used for comparison between groups. The categorical variables were expressed as quantity and percentage, and the comparison between the two groups was performed by Pearson *chi* square test or Fisher’s exact test. A two-side *p* < 0.05 was considered statistically significant.

## Results

### Baseline characteristics

According to the inclusion and exclusion criteria, 111 patients with hypertensive intracerebral hemorrhage combined with cerebral hernia were included, including 60 patients underwent endoscopic surgery and 51 patients underwent craniotomy (Table 1). There was no significant difference between the two groups in terms of baseline characteristics, preoperative GCS score, laboratory test results, and location of cerebral hemorrhage (*p* > 0.05). There was no significant difference between the two groups in the time from onset to surgical treatment (*p* > 0.05).

**Table 1:** Baseline characteristics of patients underwent hematoma evacuation.

Characteristics	NEHE group (*n* = 60)	CHE group (*n* = 51)	t/X^2^/u value	*p* value
Age (yrs)	62.0 ± 14.4	58.2 ± 11.3	1.521	0.131
Male (n, %)	46 (76.7)	35 (68.6)	0.903	0.342
Smoking (n, %)	23 (38.3)	14 (27.5)	1.469	0.226
Alcohol (n, %)	17 (28.3)	12 (23.5)	0.330	0.566
Diabetes (n, %)	9 (15.0)	6 (17.8)	0.247	0.619
Prior Stroke (n, %)	5 (8.3)	7 (13.7)	0.831	0.362
BMI (kg/m^2^)	24.9 ± 4.7	24.5 ± 4.9	0.438	0.662
SBP (mmHg)	183.0 (158.0, 207.3)	195.0 (165.5, 215.5)	1.178	0.239
WBC (10^9^/L)	14.2 ± 4.6	14.8 ± 5.0	0.619	0.537
Hemoglobin (g/L)	135.0 (124.8, 148.3)	132.0 (119.5, 150.0)	0.334	0.738
Total cholesterol (mmol/L)	4.7 ± 1.1	4.4 ± 1.3	1.158	0.249
Triglyceride (mmol/L)	1.18 (0.89, 1.89)	1.33 (0.80, 2.09)	0.154	0.878
GCS before surgery				
≤8 (n, %)	49 (81.7)	46 (90.2)	1.626	0.202
9 (n, %)	11 (18.3)	5 (9.8)		
Hematoma location (n, %)			2.066	0.356
Cerebral cortex	16 (26.7)	8 (15.7)		
ICBG	36 (60.0)	34 (66.7)		
Thalamus	8 (13.3)	9 (17.6)		
Hematoma volume (ml)	70 (60, 80)	70 (60, 83)	0.479	0.632
Ventricular hemorrhage (n, %)	39 (65.0)	30 (58.8)	0.447	0.504

ICBG, Internal capsule and basal ganglion.

### Comparison of surgical efficacy and complications

The hematoma clearance rate (%) of patients in NEHE group and CHE group was 97.65 (92.75, 100.00) and 95.00 (90.00, 100.00), respectively, with no significant difference between the two groups (p > 0.05). The operation time and intraoperative bleeding volume of patients in NEHE group were significantly less than those in the control group (*p* < 0.05). There was no significant difference in the volume of residual hematoma and in the incidence of recurrent postoperative bleeding between the two groups (*p* > 0.05). And no recurrent postoperative bleeding occurred in patients underwent ultra-early hematoma evacuation. A patient in NEHE group was transferred to CHE due to recurrent postoperative bleeding of 40 mL. The incidence of massive cerebral infarction in NEHE group was lower than that in CHE group, but the difference was not statistically significant (*p* > 0.05). There was no significant difference in the rates of pulmonary infection, gastrointestinal bleeding, and tracheotomy between the two groups (Table 2). No intracranial infection occurred in all patients.

**Table 2:** Comparison of surgical efficacy and complications.

Characteristics	NEHE group (*n* = 60)	CHE group (*n* = 51)	t/X^2^/u value	*p* value
Onset to operation time (min)	420 (330, 540)	375 (310, 432)	1.666	0.096
Operation time (min)	145.8 ± 39.9	169.4 ± 56.0	2.583	0.011
Blood loss (mL)	100 (50, 200)	600 (350, 800)	8.400	<0.001
Post-operation residual hematoma (mL)	2 (0, 5)	5 (0, 7.5)	1.243	0.214
Major ICS (*n*, %)	3 (5.0)	7 (13.7)	1.607	0.205
Complication (*n*, %)				
Recurrent ICH	19 (31.7)	17 (33.3)	0.035	0.852
Pulmonary infection	54 (90.0)	48 (94.1)	0.196	0.658
Gastrointestinal bleeding	12 (20.0)	12 (23.5)	0.203	0.653
Tracheotomy	45 (75.0)	39 (76.5)	0.032	0.857

### Outcomes

The treatment results of the two groups were compared (Table 3). The length of stay in ICU of patients in NEHE group was significantly shorter than that in CHE group (*p* < 0.05). The GOS score in NEHE group was significantly higher than that in CHE group 3 months after operation (p < 0.05). The vegetative state rate (7/60, 11.7%) and severe disability rate (19/60, 31.7%) in NEHE group were lower than those in CHE group (10/51, 19.6% and 21/51, 41.2%) respectively, but the difference was not statistically significant (*p* > 0.05). In NEHE group, 2 cases died of pulmonary infection after discharge. Seven patients died in the CHE group, including 2 patients with large amount of recurrent postoperative bleeding, 3 patients with secondary massive cerebral infarction, 1 patient with secondary brain stem hemorrhage after surgery, and 1 patient with brain swelling after secondary surgery.

**Table 3:** Follow-up outcomes.

Outcomes	NEHE group (*n* = 60)	CHE group (*n* = 51)	t/X^2^/u value	*p* value
GOS 3-month post-operation	3.3 ± 1.0	2.8 ± 1.1	2.235	0.021
NIHSS	13.0 (5.5, 30.3)	10.0 (4.0, 32.0)	0.743	0.458
ADL	45.0 (0.0, 86.3)	10.0 (0.0, 62.5)	1.734	0.083
Death (n, %)	2 (3.3)	7 (13.7)	2.723	0.099
Vegetative state (n, %)	7 (11.7)	10 (19.6)	1.340	0.247
Severe disability (n, %)	19 (31.7)	21 (41.2)	1.082	0.298
Hospital stay (d)	33.0 (25.0, 55.0)	38.0 (20.0, 59.5)	0.071	0.943
ICU stay (d)	5.5 (2.8, 9.0)	9.0 (5.0, 13.5)	3.083	0.002

## Discussion

Neuroendoscopic treatment of hypertensive intracerebral hemorrhage has been supported by many studies. Compared with traditional craniotomy, neuroendoscopic treatment has significant benefits. However, due to the severe condition, few patients of hypertensive intracerebral hemorrhage combined with cerebral hernia were treated with neuroendoscopy. The results of this study show that neuroendoscopy has the same hematoma clearance rate as craniotomy in the treatment of hypertensive intracerebral hemorrhage with cerebral hernia, but the operation time, intraoperative hemorrhage, and the incidence of massive cerebral infarction are less. Moreover, the 3-month follow-up results showed that the outcome (mortality, rate of vegetative state, and severe disability) of patients treated with neuroendoscopy was not inferior to that of patients treated with craniotomy. In general, the results of this study show that neuroendoscopy can be safely and effectively used to treat patients with hypertensive intracerebral hemorrhage combined with cerebral hernia.

Hypertension is the most important cause of cerebral hemorrhage, especially uncontrolled hypertension. Previous studies have shown that hypertensive patients have an increased risk of cerebral hemorrhage of 3.5- to 9-fold compared with people with normal blood pressure (16, 17). However, at present, the situation of hypertension in the world is not optimistic, especially in China, where the rate of blood pressure reaching the treatment goal (systolic blood pressure < 140 mmHg) is only 9% (18). Therefore, there are a large number of patients with current and potential hypertensive intracerebral hemorrhage. At present, there are still many disputes on the treatment of hypertensive intracerebral hemorrhage, especially for most supratentorial intracerebral hemorrhage, the effectiveness of surgery is still unclear (19, 20). At present, conservative treatment is often adopted for those with less hematoma, and stereotactic drainage and injection of urokinase are also used to dissolve the hematoma (19, 20). However, for patients with a large amount of hematoma and progressive deterioration of the patient’s condition, especially those with brain hernia, surgical treatment is still needed to save lives (21). For patients with cerebral hernia, the conventional surgical measures are craniotomy with bone flap and removal of hematoma under microscope. Because of the relatively large surgical trauma, the traction of brain tissue causes severe postoperative edema in the surgical area, and in addition, the secondary cerebral infarction may occur, which often requires decompressive craniectomy (22). After decompressive craniectomy, because of the lack of skull protection at the bone window, the brain tissue is dragged and swayed, which aggravates the formation of softening focus and is more likely to induce epilepsy (23, 24). In the later stage, the defect needs to be repaired again, causing secondary injury, which also increases the economic burden of patients.

Neuroendoscopic treatment of intracerebral hemorrhage has the advantages of small trauma, high safety, fast recovery, and low cost (25). Neuroendoscopic removal of intracranial hematoma has been widely carried out, but most of them are used to treat patients with relatively small hematoma and those without brain hernia (26, 27). The results of this study show that it is safe and effective to treat hypertensive intracerebral hemorrhage with cerebral hernia with neuroendoscopy. We believe that the effectiveness and safety of this treatment strategy are related to the following points: First, the hematoma was effectively cleared under direct vision. Our results showed that there was no significant difference in the hematoma clearance rate between endoscopic and craniotomy. Second, the operation time is significantly shorter than that of craniotomy, which also shortens the time of brain hernia. The key to the treatment of brain hernia is to clear the hematoma as soon as possible and alleviate the brain hernia. The neuroendoscopic surgery takes a shorter time from making a skin incision to removing the hematoma under the endoscope when compared with traditional craniotomy. The shorter the duration of brain hernia, the smaller the secondary damage (28). Third, neuroendoscopic surgery is minimally invasive. The channel with a diameter of only about 2.0 cm can be used to remove the hematoma under endoscope, which will cause less traction damage to the cortex and brain tissue when compared with traditional craniotomy. Moreover, the endoscope can directly reach the deep part of the hematoma, and the angle mirror can better observe the surrounding hematoma without too large swinging channels and pulling the brain tissue. The microscope is a columnar field of vision. For better exposure of deep hematoma and surrounding hematoma, the cortex should be stretched apart which might cause more secondary damage (29). Fourth, the intraoperative bleeding is small. Most of the bleeding in hypertensive intracerebral hemorrhage surgery comes from the head incision skin flap and the bone window edge, and there is little bleeding from the clearance of the hematoma (30). Small bone window and small incision can reduce the bleeding area, and shorten the operation time can also reduce the bleeding time.

The results of this study showed that for patients with unilateral mydriasis or bilateral mydriasis whose pupil retraction was normal before operation, the incidence of secondary massive cerebral infarction after operation was not high using microscope or endoscope (there were 7 patients with craniotomy and 3 patients with neuroendoscopy group in this study). Three patients with massive cerebral infarction after neuroendoscopic surgery was treated conservatively, and one patient underwent decompressive craniectomy. After that, the patient’s condition was stable and gradually recovered. The 3-month follow-up results showed that, compared with craniotomy, the rate of vegetative state and the rate of severe disability in patients undergoing endoscopic neurosurgery had a downward trend, while the rate of mild disability had an upward trend, but none of them reached statistical significance. First, it may be related to the small sample size. When the sample size increases, these differences will possibly show statistical significance. As mentioned earlier, clearing hematoma and reducing brain hernia in a shorter time may help reduce the rate of vegetative state and the rate of severe disability. However, it may be difficult to have a significant impact on the disability caused by irreversible damage to brain tissue (31, 32). Third, neuroendoscopic hematoma removal also has its difficulties, mainly due to the difficulty of hemostasis. In clinical practice, we found that most of the responsible blood vessels and other broken small blood vessels can be found after endoscopic aspiration of hematoma in the emergency operation of hypertensive cerebral hemorrhage with cerebral hernia. We speculate whether it is related to the rupture of new blood vessels in the process of hematoma enlargement and expansion. Some of the broken ends of blood vessels were wrapped by blood clots, but most of them still had slight bleeding after the hematoma was removed. Bipolar electrocoagulation is difficult to be implanted due to the small endoscopic channel. We usually use unipolar electrocoagulation combined with an aspirator to stop bleeding, and specifically, we use fluid gelatin or gelatin sponge to stop tiny bleeding on the wound surface. Through experience, most bleeding has been effectively stopped. In this study, there were 5 cases with recurrent postoperative bleeding volume greater than 15 mL in the endoscopic group, and only 1 case with 40 mL rebleeding was transferred to successful craniotomy. The potential reasons of recurrent postoperative bleeding include the original rupture of culprit vessel, surgery-related injury of vessels around the hematoma, the decompression of tissues around the hematoma, and uncontrolled blood pressure. Whether neuroendoscopy can increase the rate of mild disability still needs further research and observation. In this study, only 6 patients received hematoma evacuation within 4 h after onset of stroke both in NEHE (10.0%) and CHE groups (11.8%). Due to small sample, we did not analyze the efficacy of neuroendoscopic hematoma evacuation on these ultra-early patients.

This study has some limitations. First, as mentioned above, the sample size of this study is small. For the results with different trends, the current sample size is not enough to draw a final conclusion, which needs further observation in future research. Second, this study is a retrospective study. Inevitably, there is a certain bias between patients with different operations, including differences in baseline data, disease conditions, doctors’ treatment plans, and nursing care during hospitalization, and even doctors’ experience may be different. Third, this study did not further analyze the efficacy and safety of neuroendoscopy in treating hypertensive intracerebral hemorrhage with cerebral hernia at different locations, nor did it analyze patients at different times of treatment. Fourth, this study failed to analyze the influence of various factors on the efficacy and safety of neuroendoscopic treatment of hypertensive intracerebral hemorrhage with cerebral hernia, such as preoperative blood pressure level, GCS score and NHISS score. In order to further observe, summarize and analyze the efficacy and safety of endoscopic treatment of hypertensive intracerebral hemorrhage with cerebral hernia, a multicenter, prospective cohort study and a randomized controlled study are needed.

## Data availability statement

The raw data supporting the conclusions of this article will be made available by the authors, without undue reservation.

## Ethics statement

The studies involving humans were approved by The Ethics Committee of the 909th Hospital, School of Medicine, Xiamen University (Approval number: L2022011). The studies were conducted in accordance with the local legislation and institutional requirements. The ethics committee/institutional review board waived the requirement of written informed consent for participation from the participants or the participants’ legal guardians/next of kin because this is a retrospective study.

## Author contributions

YZ, XZ, and WW: conception and design. JW and LF: administrative support. WH and JL: provision of study materials or patients. YZ, XZ, JW, and FL: collection and assembly of data. YZ and XZ: data analysis and interpretation. YZ, XZ, JW, LF, WH, JL, FL, and WW manuscript writing. All authors contributed to the article and approved the submitted version.
